# Enzymes involved in the anaerobic degradation of phenol by the sulfate-reducing bacterium *Desulfatiglans anilini*

**DOI:** 10.1186/s12866-018-1238-0

**Published:** 2018-08-29

**Authors:** Xiaoman Xie, Nicolai Müller

**Affiliations:** 1 Department of Biology, University of Konstanz, Constance, Germany; 2 Konstanz Research School Chemical Biology, Constance, Germany

**Keywords:** Phenol degradation, Sulfate-reducing bacterium, *Desulfatiglans anilini*, Phenylphosphate synthase, Phenylphosphate carboxylase

## Abstract

**Background:**

The sulfate-reducing bacterium *Desulfatiglans anilini* can grow with phenol as sole source of carbon and energy under strictly anaerobic, sulfate-reducing conditions. In the nitrate-reducing bacterium *Thauera aromatic*a, the enzymes involved in phenol degradation have been well elucidated, whereas the anaerobic phenol degradation pathway by *D. anilini* was not studied in detail yet.

**Results:**

The pathway of anaerobic phenol degradation by the sulfate-reducing bacterium *Desulfatiglans anilini* was studied by identification of genes coding for phenylphosphate synthase (encoded by *pps* genes) and phenylphosphate carboxylase (encoded by *ppc* genes) in the genome of *D. anilini*, by analysis of the transcription and translation of *pps-ppc* genes, and by measurement of phenylphosphate synthase activity in cell-free extracts of phenol-grown cells. The majority of genes involved in phenol degradation were found to be organized in one gene cluster. The gene cluster contained genes *ppsα* (phenylphosphate synthase alpha subunit), *ppsβ* (phenylphosphate synthase beta subunit), *ppcβ* (phenylphosphate carboxylase beta subunit), as well as 4-hydroxybenzoyl-CoA ligase and 4-hydroxylbenzoyl-CoA reductase-encoding genes. The genes *ppsγ* (phenylphosphate synthase gamma subunit), *ppcα* (phenylphosphate carboxylase alpha subunit) and *ppcδ* (phenylphosphate carboxylase delta subunit) were located elsewhere in the genome of *D. anilini*, and no obvious homologue of *ppcγ* (phenylphosphate carboxylase gamma subunit) was found in the genome. Induction of genes *pps* and *ppc* during growth on phenol was confirmed by reverse transcription polymerase chain reaction. Total proteome analysis revealed that the abundance of enzymes encoded by the gene cluster under study was much higher in phenol-grown cells than that in benzoate-grown cells. In in-vitro enzyme assays with cell-free extracts of phenol-grown cells, phenylphosphate was formed from phenol in the presence of ATP, Mg^2+^, Mn^2+^, K^+^ as co-factors.

**Conclusions:**

The genes coding for enzymes involved in the anaerobic phenol degradation pathway were identified in the sulfate-reducing bacterium *D. anilini.* The results indicate that the first steps of anaerobic phenol degradation in *D. anilini* are phosphorylation of phenol to phenylphosphate by phenylphosphate synthase and carboxylation of phenylphosphate by phenylphosphate carboxylase.

**Electronic supplementary material:**

The online version of this article (10.1186/s12866-018-1238-0) contains supplementary material, which is available to authorized users.

## Background

Phenol is an important industrial commodity used as a precursor for the production of plastics, polycarbonates, epoxies, detergents and pharmaceutical drugs. Its wide use and toxicity has caused serious contaminations of waters and soils. Biological phenol degradation is an economic and effective method to deal with these contaminants without causing secondary pollution problems [1].

**Fig. 1:**
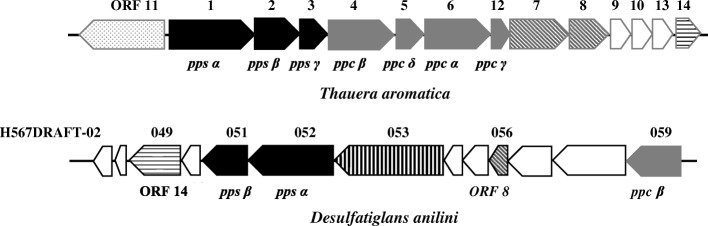
Organization of genes encoding phenylphosphate synthase (ppsαβγ) and phenylphosphate carboxylase (ppcαβγδ) in *T. aromatica* and the proposed anaerobic phenol degradation gene cluster in *D. anilini*. The directions of transcription are indicated by arrows. Similar ORFs are presented by the same shades and patterns

Anaerobic phenol degradation has been studied in detail with the denitrifying bacterium *Thauera aromatica.* Initially, phenol is phosphorylated to phenylphosphate [2], which is subsequently carboxylated to 4-hydroxybenzoate [3, 4]. The enzyme involved in the first reaction is phenylphosphate synthase (encoded by *pps* genes), which converts phenol and ATP to phenylphosphate, AMP, and phosphate [5]. Phenylphosphate synthase contains three subunits (α, β, γ), and their encoding genes are located adjacent to each other in one operon (Fig. 1). The α-subunit (70 kDa) containing a conserved histidine residue alone can catalyze the exchange of free [^14^C] phenol and the phenol moiety of phenylphosphate, but not the phosphorylation of phenol. The β-subunit (40 kDa) is required in the phosphorylation of phenol, which can transfer a diphosphoryl group to the conserved histidine residue in the α-subunit [6]. The reaction is stimulated by the addition of γ-subunit (24 kDa), but the exact function of the γ-subunit is unknown [5].

The enzyme involved in the subsequent carboxylation of phenylphosphate with CO_2_ to 4-hydroxybenzoate is phenylphosphate carboxylase (encoded by *ppc* genes), which consists of four subunits (α, β, γ, δ) [3, 4, 7]. The exchange of ^14^CO_2_ and the carboxyl group of 4-hydroxybenzoate was catalyzed by three of the subunits (α, β, γ; 54, 53, and 10 KDa). Phenylphosphate carboxylation was restored when the δ subunit (18 KDa) was added [7]. The δ subunit is assigned to the hydratase/phosphatase protein family and can catalyze alone a very slow hydrolysis of phenylphosphate. The genes coding for these four subunits are located adjacent to each other in one operon (Fig. 1). K^+^ and divalent metal cations (Mg^2+^ or Mn^2+^) are required for phenylphosphate carboxylase activity, and oxygen is an inhibitor for phenylphosphate carboxylase activity. 4-hydroxybenzoate is then catalyzed by 4-hydroxybenzoate CoA ligase to 4-hydroxybenzoyl CoA [8], which is converted to the central intermediate benzoyl-CoA by 4-hydroxybenozyl-CoA reductase [9].

The same phenol degradation pathway was proposed earlier for the iron-reducing bacterium *Geobacter metallireducens* GS-15 [10]*.* The initial steps of phenol degradation in *G. metallireducens* are accomplished by phenylphosphate synthase and phenylphosphate carboxylase as known from *Thauera aromatica*. The phenol induced gene cluster (*pps-ppc*) was identified in the genome of *G. metallireducens*, which revealed some differences compared to the corresponding gene cluster in *T. aromatica*: it is not induced specifically in phenol-grown cells and it only contains a *ppc β* homologue. In the fermenting bacterium *Sedimentibacter hydroxybenzoicus*, phenol is most likely carboxylated by an ATP-dependent 4-hydroxybenzoate decarboxylase [11, 12]. In a newly isolated strain of the sulfate-reducing bacterium *Desulfatiglans anilini*, the phenol degradation pathway appears to be the same as in *Thauera aromatica* [13]*.* Yet, phenol degradation was not studied at the biochemical and proteome level before in sulfate reducing bacteria. In the present study, we identified the catabolic enzymes and their genes involved in anaerobic degradation of phenol in the sulfate reducing bacterium *Desulfatiglans anilini*.

## Results

### Anaerobic growth with phenol or benzoate

**Fig. 2:**
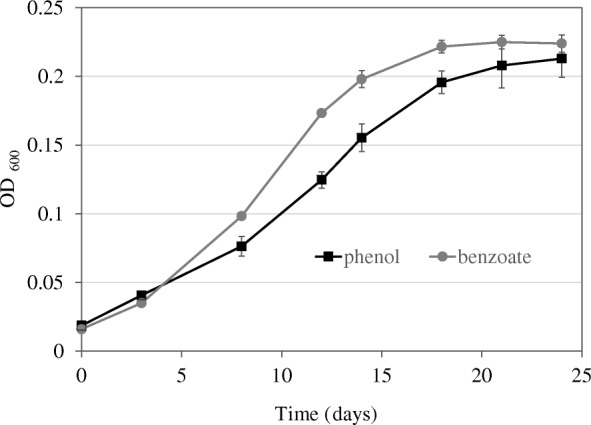
Anaerobic growth of *D. anilini* with 2 mM phenol or 2 mM benzoate plus sulfate (10 mM) as electron acceptor

The growth of *Desulfatiglans anilini* on phenol or benzoate was investigated. 2 mM phenol or 2 mM benzoate was supplied to *D. anilini* cultures as the only source of electrons with Na_2_SO_4_ as the electron acceptor (Fig. 2). The doubling time of *D. anilini* on benzoate is 4.4 days, which is slightly shorter than that on phenol (6.6 days). Culture samples were taken at different time points, and metabolites were analyzed by HPLC. No intermediate organic degradation products were detected in the growth medium. 2 mM phenol or benzoate was consumed in around 20 days along with approximately 8 mM Na_2_SO_4_ being reduced. Cells of late logarithmic phase cultures that had been pre-grown for 10 generations on the respective substrates were harvested and used for the following experiments.

### Similarity search of genes involved in phenol metabolism

The enzymes reported to be involved in phenol degradation by *T. aromatica* (ppsα, ppsβ, ppsγ, ppcα, ppcβ, ppcγ, ppcδ, ORF7–11 and ORF13–14) [14, 15] or *G. metallireducens* (BamQ, BamA, BamR and BamB) were used for a similarity search in the genome of *D. anilini* by IMG/M blast or NCBI blast. As shown in Table 1, the proteins with locus tags H567DRAFT_02052, H567DRAFT_02051, H567DRAFT_02059, H567DRAFT_02049 and H567DRAFT_02056 displayed the highest identities of 38–56% to *ppsα, ppsβ, ppcβ, ORF14 and ORF8* of *T. aromatica* individually. The genes coding for the above-mentioned proteins are located in one gene cluster, indicating that this gene cluster could be involved in phenol degradation. However, the proteins that showed highest similarities to the genes *pps*γ, *ppcδ* and *ppcα* in *T. aromatica* were the acetoin utilization protein AcuB (locus tag H567DRAFT_03126), KDO 8-P phosphatase (locus tag H567DRAFT_00862) and phenylphosphate carboxylase beta subunit (locus tag H567DRAFT_03563) individually, whose encoding genes are not located in the same gene cluster. Genome analysis did not reveal obvious homologues of *ppcγ*. For the ORFs 7, 9, 10, 11, 13, the genes showing high similarities were not localized in this gene cluster.

**Table 1:** Identities of putative genes involved in phenol or benzoate-degradation

Enzymes in *T. aromatica* or *G. metallireducens* ^a^	Annotation from IMG in *D. anilini*	Gene locus (H567DRAFT_) ^b^	Mol. weight (kDa)	Sequence coverage (%) ^c^	Score ^d^	Identities (%) ^e^
ppsα	pyruvate, water dikinase	02052	70.027	49.1	323.31	42
ppsβ	pyruvate, water dikinase	02051	39.928	21.2	93.396	38
ppsγ	acetoin utilization protein AcuB	03126	25.893	35.4	202.27	25
ppcβ	phenylphosphate carboxylase beta subunit	02059	52.24	68.2	323.31	49
ppcδ	3-deoxy-D-manno-octulosonate 8-phosphate phosphatase (KDO 8-P phosphatase)	00862	–	–	–	46
ppcα	phenylphosphate carboxylase beta subunit	03563	51.922	12.9	76.233	33
ORF 14	phenylacetate--CoA ligase	02049	50.501	9	68.567	39
ORF 8	4-hydroxy-3-polyprenylbenzoate decarboxylase	02056	–	–	–	56
BamQ^*^	6-hydroxycyclohex-1-ene-1-carboxyl-CoA dehydrogenase	01120	39.75	42.1	282.42	49
BamA^*^	6-ketocyclohex-1-ene-1-carbonyl-CoA hydrolase	01121	42.886	72.7	323.31	51
BamR^*^	cyclohexa-1,5-diene-1-carbonyl-CoA hydratase	01122	28.001	55.7	323.31	37
BamB^*^	tungsten-dependent benzoyl-CoA reductase subunit bamB	00366	75.838	46.1	323.31	80

^a^Abbreviations of the enzymes involved in phenol or benzoate (*) anaerobic degradation pathways in *T. aromatica* or *G. metallireducens* (*). ^b^IMG gene locus tag from the genome of *D. anilini*. ^c^Sequence coverage represents the extent of peptides obtained during MS-MS identification of respective protein in the total proteome analyses. ^d^The protein score from an MS/MS search is derived from the ions scores. Score and sequence coverage of the peptide finger print match as indicated by the MASCOT-search engine. ^e^The highest identity of protein sequence in *D. anilini* to that in *T. aromatica* or *G. metallireducens*. -: Not found in total proteome analyses result

For the downstream metabolism of phenol, the degradation pathway of benzoyl-CoA consists of benzoyl-CoA reductase (BamB-I) [16, 17], a cyclohexadienoyl-CoA hydratase (BamR) [18], a hydroxyenoyl-CoA dehydrogenase (BamQ) [16], and an oxoenoyl-CoA hydrolase (BamA) [19]. The genes coding for proteins which display the highest similarities to *BamQ, BamA* and *BamR* of *G. metallireducens* are located adjacent to each other in the genome of *D. anilini*.

### Reverse transcription PCR analysis

**Fig. 3:**
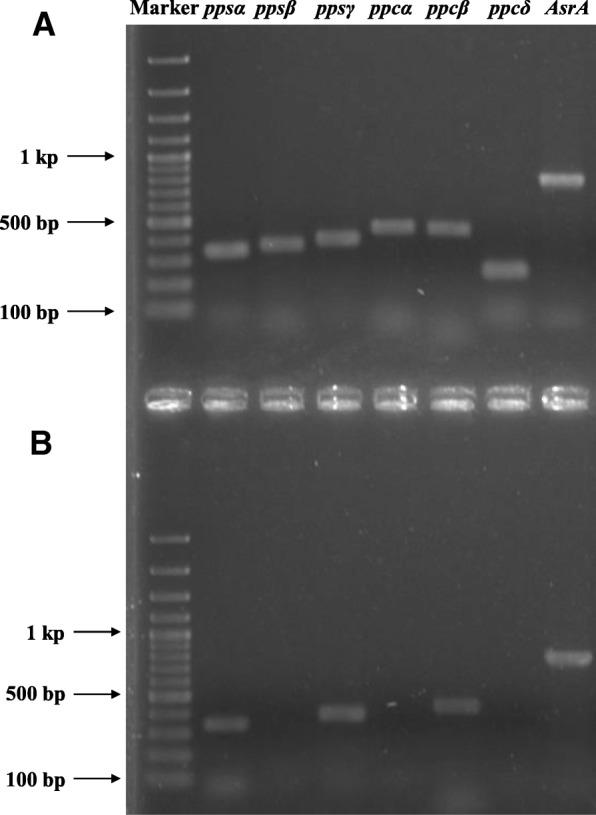
Reverse transcriptase PCR of genes *ppsα, ppsβ, ppsγ, ppcα, ppcβ and ppcδ* in *D. anilini*. (**A**) Reverse transcriptase PCR products of *ppsα, ppsβ, ppsγ, ppcα, ppcβ, ppcδ* and *AsrA* (dissimilatory adenylylsulfate reductase alpha subunit precursor; housekeeping gene) using the cDNA from phenol-grown cells as template. (**B**) Reverse transcriptase PCR products of *ppsα, ppsβ, ppsγ, ppcα, ppcβ, ppcδ* and *AsrA* (dissimilatory adenylylsulfate reductase alpha subunit precursor; housekeeping gene) using the cDNA from benzoate-grown cells as template

Reverse transcription polymerase chain reaction (RT-PCR) experiments were performed with mRNA extracted from cells grown on phenol or benzoate (Fig. 3) to test whether the putative genes of *ppsα*, *ppsβ*, *pps*γ, *ppcα*, *ppcβ*, and *ppcδ* in the genome of *D. anilini* were induced by phenol. With cDNA from phenol-grown cells, all genes under study were amplified. With cDNA from benzoate-grown cells, no amplifications of *ppsβ*, *ppcα* and *ppcδ* were obtained. This result indicates that transcription of all genes (*ppsα*, *ppsβ*, *pps*γ*, ppcα*, *ppcβ*, and *ppcδ*) was induced in phenol-grown cells, but not all of them were induced in benzoate-grown cells. Hence, this result confirmed the involvement of these genes in phenol degradation by *D. anilini*. In a control PCR experiment, genomic DNA instead of cDNA from mRNA was used as the template, and DNA fragments of the expected sizes were obtained in all cases (Additional file 1: Figure S1). The gene coding for dissimilatory adenylylsulfate reductase alpha subunit precursor was transcribed in both phenol-grown and benzoate-grown cells and used as the house-keeping gene reference.

### Total proteomics analysis

**Fig. 4:**
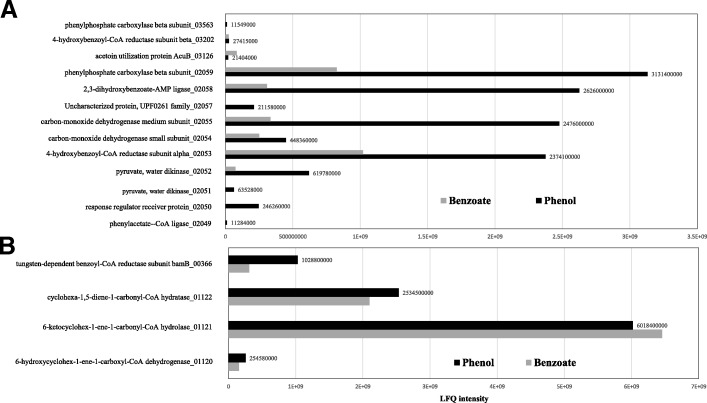
Total proteome analysis of cell-free extracts from the cells of *D. anilini* grown on phenol (black bars) and benzoate (grey bars). (**A**) label-free quantification intensity (LFQ-intensity) of the enzymes encoded by *pps-ppc* gene cluster (locus tag 02049–02059) and putative *ppsγ* (locus tag 03126) and ppcα (locus tag 03563) in *D. anilini* grown with phenol or benzoate. (**B**) LFQ-intensity of enzymes (6-hydroxycyclohex-1-ene-1-carboxyl-CoA dehydrogenase, 6-ketocyclohex-1-ene-1-carbonyl-CoA hydrolase, cyclohexa-1, 5-diene-1-carbonyl-CoA hydratase, tungsten-dependent benzoyl-CoA reductase subunit bamB) involved in benzoyl CoA metabolism in *D. anilini* grown with phenol or benzoate. The LFQ intensity value of the enzymes from phenol-grown cells are labeled at the end of the columns. The lowest LFQ intensity value observed in total proteomics analysis was 109,120, and the highest LFQ intensity observed is 16,493,000,000

To further verify the expression of putative genes involved in phenol degradation by *D. anilini*, the proteome of phenol-grown cells was compared to that of benzoate-grown cells by total proteomics analysis. Equal amounts of protein from phenol-grown cells or benzoate-grown cells were analyzed, and all protein abundances were quantified by label-free protein quantification (LFQ) (Fig. 4). The LFQ of enzymes encoded by the putative phenol degradation gene cluster (locus tag from H567DRAFT_02049 to H567DRAFT_02059) in phenol-grown cells were much higher than that in benzoate-grown cells (Fig. 4a). The gene cluster under study is a 14.4 kb gene cluster in the genome of *D. anilini*. The putative gene *ppcα* (locus tag H567DRAFT_03563) which is not located in this gene cluster was specifically induced in phenol-grown cells. Nevertheless, the putative gene *ppsγ* (locus tag H567DRAFT_03126) did not exhibit higher abundance in phenol-grown cells than in benzoate-grown cells. These results further verified the participation of the gene cluster in phenol degradation. Hence, phenol is presumed to be degraded through a similar pathway as in *T. aromatica.*

The total proteome data of phenol-grown cells and benzoate-grown cells also demonstrate the existence of the putative enzymes to be involved in anaerobic benzoyl-CoA degradation. The label-free quantification (LFQ) intensity of these enzymes (H567DRAFT_01120 6-hydroxycyclohex-1-ene-1-carboxyl-CoA dehydrogenase, H567DRAFT_01121 6-ketocyclohex-1-ene-1-carbonyl-CoA hydrolase, H567DRAFT_01122 cyclohexa-1, 5-diene-1-carbonyl-CoA hydratase) did not show significant differences between phenol-grown cells or benzoate –grown cells (Fig. 4b).

### Phenylphosphate synthase activity

**Fig. 5:**
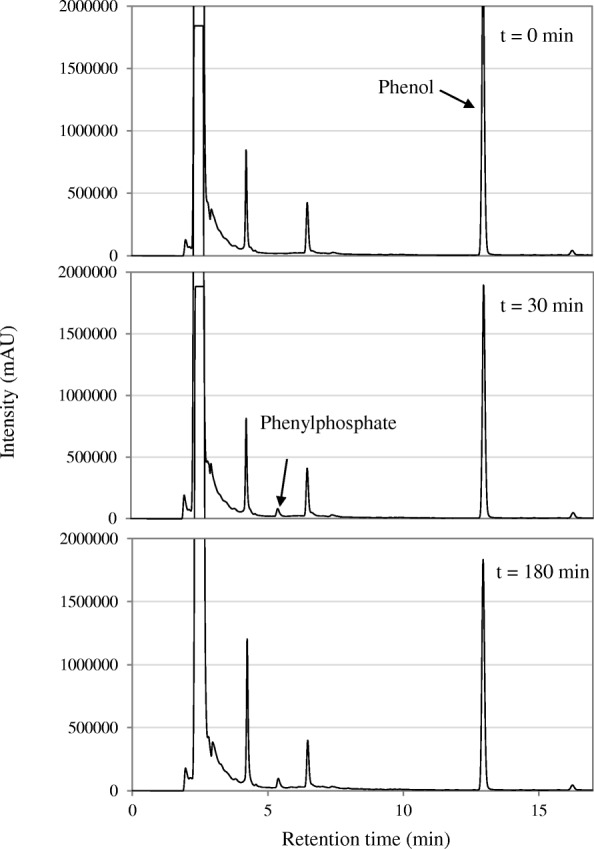
Time course of enzymatic phenylphosphate formation from phenol and ATP by the putative phenylphosphate synthase discontinuously assayed by HPLC with crude cell-free extract as sample

In-vitro enzyme activity assays were carried out with cell-free extracts of phenol- or benzoate-grown cells. The enzyme activity of phenylphosphate synthase was observed by detecting the formation of phenylphosphate over time, using phenol and ATP as the substrates with cell-free extracts of phenol-grown cells. Fig. 5 shows that these cell-free extracts were able to convert phenol to phenylphosphate with ATP as a co-substrate and Mg^2+^, Mn^2+^, and K^+^ as cofactors. Extracts of phenol-grown *T. aromatica* cells catalyzed the MgATP-dependent formation of [^14^C] phenylphosphate from [U-^14^C] phenol at a specific rate of 1.5 nmol min^− 1^ mg^− 1^ of protein [5]. In our study, the rate of phenylphosphate formation catalyzed by phenylphosphate synthase was tested as 0.52 nmol min^− 1^ mg^− 1^ of protein which is lower than that of *T. aromatica* cells. The activity was inhibited by oxygen, and no activity was measured with cell free extracts of benzoate grown cells.

### Phenylphosphate carboxylase activity

The activity of phenylphosphate carboxylase was assayed either by spectrophotometric assays or HPLC [3]. In spectrophotometric assays no change of absorption could be measured as a consequence of phenylphosphate consumption at 235 nm or 4-hydroxybenzoate production at 280 nm. No substrate consumption or product formation was observed by HPLC, neither with CO_2_ nor with CO as co-substrate.

## Discussion

**Fig. 6:**
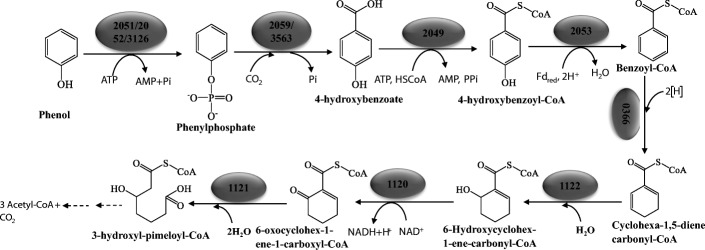
Proposed pathway of anaerobic phenol degradation by *D. anilini.* Numbers in dark ellipses indicate the locus tags of the enzymes

In the present study, the initial steps of phenol activation in a sulfate-reducing bacterium were studied by characterizing the transcription and expression of *pps-* and *ppc-* like ORFs and in-vitro phenylphosphate synthase assays. The results revealed that the phenol degradation pathway in this sulfate-reducing bacterium *D. anilini* (Fig. 6) is analogous to the known phenol degradation pathway in the nitrate-reducing bacterium *T. aromatica.*

The first step for phenol activation in *D. anilini* is a phosphorylation of phenol to phenylphosphate catalyzed by phenylphosphate synthase. Homologues of the three genes coding for the three subunits of phenylphosphate synthase (*ppsαβγ*) in *T. aromatica* are present in the genome of *D. anilini*. The genes *ppsα* (locus tag H567DRAFT_02052) and *ppsβ* (locus tag H567DRAFT_02051) are two adjacent genes showing homologies to pyruvate, water dikinase, and are transcribed and translated during growth on phenol. The putative gene *ppsγ* (locus tag H567DRAFT_03126) was transcribed both in phenol- and in benzoate-grown cells. The expression of the putative gene *ppsγ*, which is not located in the same gene cluster with *ppsα* and *ppsβ*, is lower in phenol-grown cells than in benzoate-grown cells. The enzyme activity of phenylphosphate synthase was observed in vitro in cell-free extracts of phenol-grown cells by measuring the conversion of phenol to phenylphosphate. Since the conversion of phenol to phenylphosphate can be accomplished without the presence of *ppsγ*, further verifications are needed to prove the participation of gene *ppsγ* in phenol degradation in *D. anilini*.

After activation of phenol to phenylphosphate, the latter is carboxylated to 4-hydroxybenzoate by phenylphosphate carboxylase. Homologues of three genes coding for the three subunits (*ppcαβδ*) of phenylphosphate carboxylase in *T. aromatica* are present in the genome of *D. anilini*. No homologue for *ppcγ* was found in the genome of *D. anilini.* The gene cluster under study carries only a homologue for the β-subunit of phenylphosphate carboxylase. The genes, *ppcα, ppcβ, ppcδ*, were all transcribed during growth on phenol. The expression of these genes except for *ppcδ* was observed in phenol-grown cells. However, attempts to demonstrate phenylphosphate carboxylase activity in cell-free extracts of phenol-grown cells failed. Earlier studies have shown, that the activity of phenylphosphate carboxylase in *Thauera aromatica* (earlier known as *Pseudomonas* strain K 172) is extremely oxygen sensitive and its activity in oxygen-saturated buffer had a half-life of 30 s [3, 7]. It is therefore likely, that phenylphosphate carboxylase is also present in *D. anilini*, but inactivated during cell-lysis despite all precautions made for excluding oxygen from the samples. The fact that all necessary enzymes for the degradation 4-hydroxybenzoate were expressed during growth with phenol leads to conclude that phenylphosphate, whose presence as an intermediate was proven, needs to undergo a carboxylation or carbonylation with phenylphosphate carboxylase as the most likely responsible enzyme.

4-hydroxybenzoate is subsequently transformed to the central intermediate benzoyl-CoA via 4-hydroxybenzoyl-CoA, the enzymes involved are 4-hydroxybenzoyl-CoA ligase and 4-hydroxybenzoyl-CoA reductase. The gene (locus tag H567DRAFT_02049) coding for 4-hydroxybenzoyl-CoA ligase in the genome of *D. anilini* shows homologies to genes coding for phenylphosphate-CoA ligase. The genes coding for 4-hydroxybenzoyl-CoA ligase and the α-subunit of 4-hydroxybenzoyl-CoA reductase are located in the gene cluster under study. This gene cluster carries a carbon monoxide dehydrogenase medium subunit (locus tag H567DRAFT_02055) and a carbon monoxide dehydrogenase small subunit (locus tag H567DRAFT_02054), which can catalyze the reduction of CO_2_ to CO. The resulting CO is combined with a methyl group to form acetyl-CoA by acetyl-CoA synthase through the Wood-Ljungdahl pathway in some anaerobic microbes [20, 21]. In *D. anilini* this enzyme system is needed for cleaving acetyl-CoA to CO and a methyl group to allow complete oxidation of the substrate to CO_2_ with sulfate as electron acceptor [22]. Since carbon monoxide dehydrogenase (CODH) was strongly expressed in phenol-grown cells in comparison to benzoate-grown cells (Fig. 4), CODH may also play a role in the upper phenol degradation pathway. For example, it could convert CO_2_ to CO and employ CO as a co-substrate to activate phenylphosphate, as reported in anaerobic acetone degradation by sulfate-reducing bacteria [23]. Another presumption is that CODH may have a reductive dehydroxylation function, by dehydroxylating 4-hydroxybenzoate or 4-hydroxybenzoyl-CoA to benzoate or benzoyl-CoA respectively. The function of 2, 3-dihydroxybenzoate-AMP ligase (locus tag H567DRAFT_02058), which is induced during growth with phenol, is unknown. In benzoate-grown cells, some of the genes in this gene cluster were still transcribed and translated. A possible reason may be that 10 generations of adaptation to benzoate are not enough to switch the metabolism from phenol to benzoate utilization completely.

The results obtained in this work support the hypothesis that sulfate-reducing bacteria use for phenol degradation a similar strategy as nitrate-reducing bacteria do. But it remains unclear how a sulfate reducer with its small energy budget can afford the high energy expenditure for phenol carboxylation and hydroxybenzoic acid activation which together require up to four ATP equivalents. The lack of *ppcγ* in the genome of *D. anilini* is in accordance with the results in the iron-reducing bacterium *Geobacter metallireducens* GS-15 [10], indicating a different catalytic mechanism of phenylphosphate carboxylase in sulfate-reducing and in iron-reducing bacteria. One possible activation mechanism that could be considered is a hypothetical phenylphosphate carbonylation to 4-hydroxybenzaldehyde with CO that is produced by the CO dehydrogenase located in the abovementioned gene cluster.

## Conclusion

In this study, the genes coding for the enzymes involved in the anaerobic conversion of phenol to benzoyl-CoA were identified in the sulfate-reducing bacterium *D. anilini*. Analysis of the genome, transcriptome and proteome revealed that phenol is most likely activated to phenylphosphate, followed by carboxylation to 4-hydroxybenzoate, which is analogous to the well-known phenol degradation pathway in nitrate-reducing bacteria. Further evidence for phenylphosphate as an intermediate of phenol degradation in *D. anilini* is provided by in-vitro enzyme assays. Activity of phenylphosphate carboxylase could not be demonstrated by in-vitro enzyme assays, however all necessary genes for this enzyme complex were specifically expressed during growth with phenol. This work contributes to completing the picture of the phenol degradation pathway in anaerobic bacteria depending on different electron acceptors.

## Methods

### Bacterial strains and culture media

*Desulfatiglans anilini* DSM 4660 was isolated from marine sediments [24] and described as a sulfate-reducing bacterium oxidizing aniline. *D. anilini* was grown in bicarbonate-buffered (30 mM) and sulfide-reduced (2 mM) brackish water medium [25]. After autoclaving at 121 °C and 1 atm overpressure and cooling to room temperature under a stream of N_2_/CO_2_, 1 mL selenite tungstate solution/L and 1 mL 7 vitamins solution/L [26] were added. A heat-sterilized stock solution of a trace element mixture (SL 13) [27] was added to the basal medium (1:1000 *v*/v). Resazurin (0.4 mg mL^− 1^) was used as redox indicator. The pH was adjusted to 7.2. Benzoate or phenol was added anoxically from filter-sterilized anoxic stock solutions to 2 mM final concentration as growth substrate plus sulfate (10 mM) as terminal electron acceptor.

### Adaptation of *D. anilini* to utilization of phenol or benzoate

*D. anilini* was inoculated into fresh medium with each 1 mM phenol or benzoate at an initial OD_600_ of around 0.04. The OD_600_ was monitored once per week until a maximum OD_600_ of around 0.18 was reached after 27 days. Cells were subsequently transferred to fresh medium four more times reaching over 135 days from initial culture.

### Reverse transcription PCR analysis

For extracting RNA of *D. anilini*, 20 mL of phenol- or benzoate-grown cells in log-phase were collected by centrifuging at 11,700 x g for 20 min. Cells were washed with ultrapure water and centrifuged again. The pellets were used to extract RNA by using the TRIzol®Reagent with the PureLink®RNA Mini Kit (Thermo Fisher Scientific, Waltham, Massachusetts, USA) according to the manufacturer’s protocol. RNA Clean & Concentrator Kit (ZYMO RESERACH, Irvine, California, USA) was used to remove contaminating DNA from RNA samples. The first strand cDNA was synthesized from total RNA using the SuperScript®III First-Strand Synthesis System for RT-PCR Kit (Invitrogen, Waltham, Massachusetts, USA) with random hexamer primers. Genomic DNA of *D. anilini* was isolated from 1.5 ml of a dense culture using the Gentra Puregene Cell Kit (Qiagen). The concentrations of DNA, RNA and cDNA were measured with a NanoDrop™ 2000c Spectrophotometer (Thermo Fisher Scientific, Waltham, Massachusetts, USA).

PCR amplification was performed using a T100 Thermal Cycler (Bio-Rad, Hercules, California, USA). The standard PCR mixture had a volume of 25 μL and contained 2.5 μL of 10 × PCR buffer, 5 nmol dNTPs, 50 pmol of each primer (Microsynth, Balgach, Switzerland), 4 nmol MgCl_2_, 0.2 μL Tag-polymerase (5 U/μL, Thermo Fisher Scientific, Waltham, Massachusetts, USA), and 2 μL cDNA or 10 to 50 ng genomic DNA as template. The PCR program consisted of an initial denaturation step at 94 °C for 3 min, followed by 31 cycles of 94 °C for 30 s, 60 °C for 30 s, and 72 °C for 1 min, and a final elongation step of 72 °C for 5 min. Primer pairs used to amplify approximately 200–500 bp fragments of genes are listed in Table 2. The PCR purification products’ qualities were analyzed by electrophoresis in a 1.0% agarose gel at 110 V for 30 min.

**Table 2:** Primers used in reverse transcription PCR analysis

PCR product	Gene locus tag	Primer sequence
*ppsα*	H567DRAFT_ 02052	ppsα-1: AAGATCCTCACCAAGCACGG
		ppsα-2: GGGGAACCCGGTGATTTCAT
*ppsβ*	H567DRAFT_ 02051	ppsβ-1: TCATGTTCTCGCTCAACCCC
		ppsβ-2: AGATCGATTCAGGGAACGGC
*ppsγ*	H567DRAFT_ 03126	ppsγ-1: CGGACAGGGATCTCAAACGG
		ppsγ-2: CGTTCGTAGCTGGTCAGGAT
*ppcα*	H567DRAFT_ 03563	ppcα-1: ACCC TGTGGCAGCAGTTATC
		ppcα-2: TAGAATCCCAGCTCCGACCA
*ppcβ*	H567DRAFT_ 02059	ppcβ-1: TGACCATGGCCGTTTCCTAC
		ppcβ-2: TCTTGACCATTTCGGGGTCG
*ppcδ*	H567DRAFT_ 00862	ppcδ-1: AAGTCGTCATCATCACGGGC
		ppcδ-2: AAATCGGCCATTTCACGGAC
*Asrα*	H567DRAFT_02821	Asrα-1: ATGACCATTTCTCAGGCGCA
		Asrα-2: GTCGCGCTTCATCATTTCCC

### Preparation of cell-free extracts

Cultures grown on phenol or benzoate were harvested at the end of the exponential growth phase (OD_600_ = 0.15–0.20) in an anoxic chamber (Coy, Ann Arbor, USA) by centrifugation (20,300 x g for 30 min at 4 °C, Dupont Sorvall, Midland, Canada). For total proteomics analysis, cells were washed twice by repeated centrifugation in anoxic 200 mL 50 mM potassium phosphate buffer containing 3 mM dithiothreitol, pH 7.5, and resuspended in 3–4 mL of the same buffer. Cells were broken anoxically by three passages through a cooled MiniCell French pressure cell (SLM Aminco, Cat. No. FA003, Urbana, Illinois, USA) operated at 137 MPa pressure. Cell debris was removed by centrifugation at 30,300 x g for 30 min at 4 °C to obtain the crude extract. The soluble protein fraction was obtained by ultra-centrifugation (150,000 x g for 60 min, Optima™ TL Ultracentrifuge, Beckman Coulter, Brea, California, USA) of the crude extract to remove insoluble membrane particles. The protein concentration was estimated with the Bradford assay using bovine serum albumin as protein standard [28].

### Total proteomics analysis and database search

The supernatants containing soluble proteins were used for total proteomics analysis, whose concentrations were 2.7 mg mL^− 1^ (phenol-grown cells) and 2.9 mg mL^− 1^ (benzoate-grown cells), from which 500 μL supernatant was submitted to peptide-fingerprinting-mass spectrometry at the Proteomics Facility of the University of Konstanz. Total proteome analysis was performed using a LTQ Orbitrap Discovery with an Eksigent 2D-nano HPLC (Thermo Fisher Scientific, Waltham, Massachusetts, USA). The mass spectrometry data was analyzed by the Mascot search engine [v2.2.2 from Matrix Science] [29], to identify and characterize proteins from the protein database of the IMG annotated genome of *D. anilini*. Quantitative analysis of the identified proteins was done by label-free quantification using the LFQ algorithms included in the Proteome Discoverer software package V1.3 (Thermo Scientific). Relative protein abundances were expressed as label-free quantification intensity (LFQ-intensity).

### Phenylphosphate synthase activity

Cell extracts from cells grown on phenol or benzoate were prepared in 50 mM imidazole-HCl buffer (pH 7.0), 0.5 mM dithiothreitol, 0.5 mg DNase I, followed with a French press and ultra-centrifugation (30 min; 30,300 x g) to remove cell debris. The standard enzyme assay mixture (2 mL) for phenylphosphate synthase contained approximately 1 mg protein, 2 mM ATP, 2 mM MgCl_2_, 2 mM MnCl_2_, 2 mM KCl and 1 mM phenol. The enzyme tests were performed at 30 °C under strictly anaerobic conditions. To analyze the enzyme product, 300 μL samples were withdrawn at different time points and the reaction was stopped by addition of an equal volume of dichloromethane and centrifuged (11,700 x g for 10 min). The supernatant was transferred into 200 μL HPLC vials and analyzed by HPLC.

### Phenylphosphate carboxylase activity

Cell extracts were prepared in 50 mM imidazole-HCl buffer (pH 7.0), 0.5 mM dithiothreitol, 10% glycerol, 0.5 mg DNase, using a French press, followed by ultra-centrifugation (30 min; 30,300 x g) to remove cell debris. The enzyme assays were performed at 30 °C under strictly anoxic conditions. The standard assay mixture (2 mL) contained 50 mM imidazole-HCl buffer (pH 7.0) with 0.5 mM dithiothreitol, 0.7 mg protein, 2 mM MgCl_2_, 2 mM MnCl_2_, 20 mM KCl, 1 mM phenylphosphate and 30 mM NaHCO_3_ (or 10% CO). The reaction products were monitored by U*V*/Vis absorption spectra or HPLC analysis. For spectrophotometric assays, 50 μL samples were withdrawn from the assay mixture at different time points, added into 950 μL of 1 M KOH solution and the absorption was determined at two wavelengths for quantification of phenylphosphate (phenolate ion at pH 14; 235 nm; ε = 9400 M^− 1^ cm^− 1^) and the product 4-hydroxybenzoate (280 nm; ε = 16,300 M^− 1^ cm^− 1^) [3]. For HPLC analysis, 300 μL samples were withdrawn from the assay mixture at different time points and the reaction was stopped by addition of an equal volume of acetonitrile and centrifuged (11,700 x g for 10 min). The supernatant (200 μL) was transferred to HPLC vials and analyzed by HPLC.

### Analytical methods

The concentrations of phenol, phenylphosphate and 4-hydroxybenzoate were determined with a reversed-phase HPLC (Shimadzu, Kyoto, Japan) system equipped with a UV-visible diode array detector and a 4 um Max-RP 80 Å LC column (250*4.6 mm, Synergi) (Phenomenex, Torrance, California, USA) at 25 °C. Eluents were prepared by mixing ultrapure water with 0.1% H_3_PO_4_ (buffer B), and acetonitrile with 0.1% H_3_PO_4_ (buffer A) and filtration through 0.2 μm. A gradient of buffer B increasing from 80 to 60% was used at a flow rate of 1 mL min^− 1^. 50 uL samples were injected into the column. The compounds were identified by comparing the retention times and UV-spectra of peaks to the retention time and UV-spectra of the respective standards. Figure 5 was prepared by exporting the chromatogram data of the 200 nm PDA-channel from the Shimadzu LC solutions software to ASCII – format (time (s) and intensity (mAU)) and the time data was converted from s to min. Then, the data was converted to a Microsoft – Excel diagram to obtain a better resolution of the chromatogram layout.

### Chemicals

All standard chemicals were of analytical quality and were obtained from Fluka (Buchs, Switzerland), Merck (Darmstadt, Germany) or Sigma (St. Louis, USA). Gases were purchased from Messer-Griesheim (Darmstadt, Germany) and Sauerstoffwerke Friedrichshafen (Friedrichshafen, Germany).

## Additional file

Additional file 1: **Figure S1.** PCR products using genomic DNA as template. (PDF 73 kb)

## Abbreviations

ASCII: American Standard Code for Information Interchange
AsrA: dissimilatory adenylylsulfate reductase alpha subunit precursor
BamA: oxoenoyl-CoA hydrolase
BamB-I: benzoyl-CoA reductase
BamQ: hydroxyenoyl-CoA dehydrogenase
BamR: cyclohexadienoyl-CoA hydratase
CODH: carbon monoxide dehydrogenase
*D. anilini*: *Desulfatiglans anilini* DSM 4660
*G. metallireducens*: *Geobacter metallireducens* GS-15
HPLC: High-performance liquid chromatography
IMG/M: Integrated Microbial Genomes & Microbiomes
LFQ: Label-free quantification intensity
MS: mass spectrometry
NCBI: National Center for Biotechnology Information
ORF: Open reading frame
ppc: phenylphosphate carboxylase
ppcα: phenylphosphate carboxylase alpha subunit
ppcβ: phenylphosphate carboxylase beta subunit
ppcγ: phenylphosphate carboxylase gamma subunit
ppcδ: phenylphosphate carboxylase delta subunit
ppsα: phenylphosphate synthase alpha subunit
ppsβ: phenylphosphate synthase beta subunit
ppsγ: phenylphosphate synthase gamma subunit
RT-PCR: Reverse transcription polymerase chain reaction
*T. aromatica*: *Thauera aromatica*; pps: phenylphosphate synthase
U*V*/Vis: Ultraviolet–visible spectroscopy
