# Supplementary figures and images for: Enzymes involved in the anaerobic degradation of phenol by the sulfate-reducing bacterium Desulfatiglans anilini
Source: BMC Microbiol. 2018 Aug 29;18:93. doi: 10.1186/s12866-018-1238-0 (PMC6114531; doi:10.1186/s12866-018-1238-0)

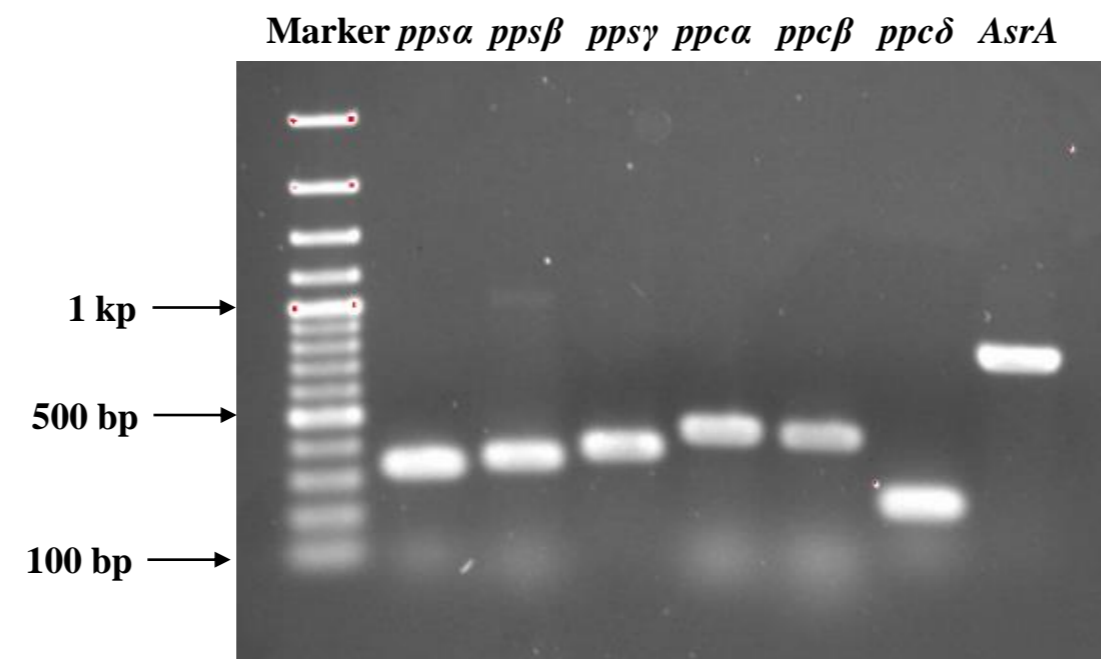

Supplement: Supplementary file 1 — Figure S1. PCR products using genomic DNA as template. (PDF 73 kb) [file 12866_2018_1238_MOESM1_ESM.pdf]
